# Association between the Planetary Health Diet Index and chronic constipation and diarrhea risk in general population: A cross-sectional analysis of NHANES

**DOI:** 10.1097/MD.0000000000045468

**Published:** 2025-11-07

**Authors:** Renwei Qi, Haoliang Zhai, Dongjia Xu, Yu Wen, Sinan Xu

**Affiliations:** a Department of Gastroenterology, Haining People’s Hospital, Jiaxing, Zhejiang, China.

**Keywords:** chronic constipation and diarrhea, diet, NHANES, the Planetary Health Diet Index

## Abstract

Diet is an important factor influencing chronic constipation and diarrhea. This investigation proposes to examine the correlation between the Planetary Health Diet Index (PHDI), a new dietary scoring system, and the risk of chronic constipation and diarrhea in the general population. Data on 13,669 adults (≥20 years) were extracted from the 2005 to 2010 National Health and Nutrition Examination Survey. The PHDI was calculated by two 24-hour dietary recall interviews and consisted of scores from 15 food groups with a total score range of 0 to 140. Multivariable logistical regression analyses were employed to examine the correlation between PHDI and chronic constipation/diarrhea, and presented as odds (OR) and 95% confidence interval. Subgroup evaluations were carried out according to population and disease characteristics. The weighted quantile sum analysis was applied to evaluate the effect of total PHDI and its components on chronic constipation. Among these 13,669 participants, 1027 reported chronic constipation, 1061 had chronic diarrhea, and 11,581 maintained normal bowels. The second (63.69–66.67) (OR = 0.73 [0.57–0.92]) and third (>66.67) tertiles (OR = 0.68 [0.54–0.85]) of PHDI were connected to lower odds of chronic constipation versus the first tertile of PHDI (<63.69). No significant correlation was identified between PHDI and chronic diarrhea (*P* > .05). There was a nonlinear link of PHDI with chronic constipation (*P*_overall_ < .001, *P*_nonlinear_ < .001), and the risk of chronic constipation in individuals decreased with the increase in PHDI. Subgroup analysis indicated that the connection between PHDI and chronic constipation risk was more obvious in populations aged <65 years, those without diabetes, and those with dyslipidemia (*P* < .05). The weighted quantile sum analysis demonstrated that the overall mixed exposure effect of PHDI on the risk of chronic constipation was 0.96 (95% confidence interval: 0.94–0.97) (*P* < .001). Among the contributions of each component to PHDI, “added sugar and fruit juices” contributed the most to the effect of PHDI, followed by “saturated oils and transfat,” while “legumes” and “nuts and seeds” contributed the least. A high PHDI is correlated with a lower risk of chronic constipation in the general population, which may suggest the potential benefits of dietary modification.

## 1. Introduction

Chronic constipation and chronic diarrhea are common intestinal disorders that affect more than 10% and 5% of the global population, respectively.^[1,2]^ Both conditions not only lead to reduced quality of life and psychological distress in patients but can also increase the risk of cardiovascular disease (CVD) and death.^[3]^ Low physical activity, substance use, gut microbiota dysbiosis, and dietary intake are all factors that influence chronic constipation/diarrhea.^[4,5]^ Evidence highlights dietary patterns as a modifiable risk factor, with low fiber intake, dehydration, and gut microbiota imbalances exacerbating constipation, while excess sugar, processed foods, or food intolerance may trigger diarrhea.^[6,7]^ However, the role of comprehensive dietary patterns in managing these conditions remains insufficiently investigated.

The Planetary Health Diet Index (PHDI), proposed by the EAT-Lancet committee, is a new dietary scoring system that emphasizes increasing the intake of plant foods and whole grains and limiting excessive intake of red meat and added sugars.^[8,9]^ The proportion of processed foods in dietary energy intake is increasing,^[10]^ and a suboptimal diet is a major cause of cardiovascular metabolic diseases.^[11]^ High PHDI scores have been reported to be significantly connected to reduced risk of CVD, metabolic abnormalities, fatty liver conditions, and all-cause mortality.^[9,12–15]^ This may be because high PHDI scores are related to improved gut microbiota diversity and reduced inflammation, factors that are closely linked to bowel function.^[16,17]^ Previous studies have presented that intake of plant-based foods such as vegetables and fruits was beneficial to gut health,^[18,19]^ but excessive fiber intake may also lead to elevated diarrhea incidence.^[20]^ Moreover, red meat and saturated fatty acid intake were positively linked to the risk of adverse gastrointestinal disorders such as constipation.^[21,22]^ However, the correlation between PHDI and chronic constipation and diarrhea is unclear.

Therefore, this investigation intended to assess the correlation between PHDI and the risk of chronic constipation and diarrhea in the general population utilizing data from a large database to provide a dietary basis for the daily management of intestinal health.

## 2. Methods

### 2.1. Study design and population

The health record data of the participants in the National Health and Nutrition Examination Survey (NHANES) dataset were employed in this cross-sectional study. NHANES is a cross-sectional survey on the health and nutritional status of nonhospitalized populations established by the National Center for Health Statistics of the United States (US) (https://wwwn.cdc.gov/nchs/nhanes/default.aspx), and it conducts the survey every 2 years. NHANES adopts a complex multistage probability sampling design based on counties, segments, households, and individuals to select the surveyed population. NHANES collected individual data in the form of interviews and physical examinations containing demographic, health-related, nutrition-related, and laboratory tests. The data for this investigation were based on the 2005 to 2010 NHANES surveys as complete stool typing data and PHDI calculation data are only available in these cycles. Participants aged ≥20 years were selected. The exclusion criteria for participants were: individuals with a diagnosis of colorectal cancer; individuals who were pregnant; individuals with extreme energy intake (<500 or >8000 kcal/d); individuals without complete data for PHDI calculation; and individuals without information on Bristol Stool Form assessment. The National Center for Health Statistics Research Ethics Review Board approved the protocols of NHANES. Before participating in the survey, each participant signed a written informed consent form.

### 2.2. Outcomes

The NHANES bowel health questionnaire (BHQ060) was applied to identify patients with chronic constipation and chronic diarrhea: “Please look at this card and tell me the number that corresponds to your usual or most common stool type.” Participants who answered type 1 (separate hard lumps, such as nuts) and type 2 (sausage-like, but lumpy) were considered to have chronic constipation, whereas participants who answered type 6 (fluffy pieces with ragged edges, a mushy stool) and type 7 (watery, no solid pieces) were considered to have chronic diarrhea.^[23,24]^ Individuals with the other types (types 3, 4, and 5) of stools were considered to have normal bowels.

### 2.3. Calculation of PHDI

The dietary intake data of the participants were collected through two 24-hour dietary recalls. Participants reported all foods and beverages consumed during the previous 24 hours. The first recall data were collected through face-to-face interviews, followed by a second interview conducted via telephone 3 to 10 days later. The diet interview room provides a set of measuring tools (e.g., various glasses, bowls, measuring cups, and spoons) for participants to report the amount of food they consumed. Dietary recall data in NHANES were categorized into 37 US Department of Agriculture food pattern components based on the food composition table. Detailed dietary interviews and data processing procedures are available at the following website: https://wwwn.cdc.gov/Nchs/Data/Nhanes/Public/2009/DataFiles/DR1IFF_F.htm. The dietary intake data utilized in this investigation were the average of the 2 dietary intake data.

PHDI was calculated using the dietary index package.^[25]^ The 15 food groups in the PHDI (whole grains, starchy vegetables, non-starchy vegetables, whole fruits, dairy food, red/processed meat, poultry, eggs, fish, nuts and seeds, legumes, soybean, saturated oils and transfat, unsaturated oils, added sugar, and fruit juices) were assigned scores ranging from 0 (lowest) to 10 (highest) based on the intake criterion (Table S1, Supplemental Digital Content, https://links.lww.com/MD/Q537), and each food group was assigned a different weighting (0.5 or 1).^[9,15]^ The total PHDI score ranges from 0 to 140. The PHDI scores in this investigation were grouped into 3 categories based on tertiles.

### 2.4. Data collection

Data on individuals were gathered incorporating age, gender, race, education, poverty-to-income ratio (PIR), marriage, current smoking, current drinking, physical activity, hypertension, diabetes, dyslipidemia, CVD, chronic kidney disease (CKD), depression, waist-to-height ratio (WHR), and total energy. Physical activity was expressed using energy expenditure (recommended metabolic equivalent [MET] × exercise time [min] for the corresponding activity [MET·min/wk]). Hypertension, diabetes, and dyslipidemia were determined on the basis of self-reported medical history, medication history, or laboratory diagnostic indicators of the corresponding disease. Depression was assessed by the Patient Health Questionnaire 9 score, and individuals with Patient Health Questionnaire 9 scores ≥10 had clinically significant depressive symptoms.^[26]^ CVD was identified on the basis of self-reported medical history or medication history. According to the 2024 Guidelines, CKD was defined as a urinary albumin-to-creatinine ratio ≥ 30 mg/g and/or an estimated glomerular filtration rate < 60 mL/min/1.73 m^2^.^[27,28]^

### 2.5. Statistical analysis

Statistical power calculation was conducted using PASS 15.0.5 software (NCSS, LLC, Kaysville). The statistical power for constipation as an outcome was 0.9999, indicating that the sample size of the current study is sufficient to support statistical analysis. NHANES weighting variables (SDMVSTRA, WTDR2D, SDMVPSU) were applied in the analysis of this investigation because of the stratified multistage sampling design of the NHANES database. For continuous data, means with standard errors were utilized; for categorical data, frequencies with percentages [n (%)] were employed. For group comparisons, the weighted variance analysis F-test was applied for continuous data and the Rao-Scott Chi-square test was employed for categorical data. Several variables (e.g., WHR with 2.25% missing data) contained missing values, which were imputed utilizing multiple interpolation methods. Comparative analysis revealed no significant differences between pre- and post-imputation results (Table S2, Supplemental Digital Content, https://links.lww.com/MD/Q537).

Confounding factors related to chronic constipation/diarrhea were initially selected through weighted univariable logistic regression, and factors with *P* < .05 were adjusted in multivariable analysis (Table S3, Supplemental Digital Content, https://links.lww.com/MD/Q537). Univariate and multivariable logistical regression were employed to examine the correlation between PHDI and chronic constipation/diarrhea. The correlation was summarized as odds ratio (OR) and 95% confidence interval (95% CI). Multivariable analyses adjusted for gender, race, education, PIR, marriage, current drinking, depression, and total energy in the analysis of chronic constipation, and age, gender, race, education, PIR, current smoking, current drinking, physical activity, hypertension, diabetes, dyslipidemia, CVD, CKD, depression, and WHR in the analysis of chronic diarrhea. Moreover, subgroup evaluations were carried out according to population and disease characteristics. The nonlinear correlation of PHDI with chronic constipation/diarrhea was explored utilizing restricted cubic spline. Furthermore, weighted quantile sum (WQS) analysis was applied to further evaluate the correlation between PHDI and chronic constipation/diarrhea. WQS is a statistical model designed to examine the contribution of individual components of a mixture to the overall effect by coding each component of the mixture by quartile and assessing the overall effect of the mixture through a weighted index. In this study, the premise assumptions of the logistic regression model, such as linearity, independence among independent variables, and goodness-of-fit tests, all meet the requirements (Figure S1, Supplemental Digital Content, https://links.lww.com/MD/Q537). Statistical analyses were carried out in R 4.4.3 software (Institute for Statistics and Mathematics, Vienna, Austria), utilizing *P* < .05 as statistically significant.

## 3. Results

### 3.1. Characteristics of participants

**Figure 1.**
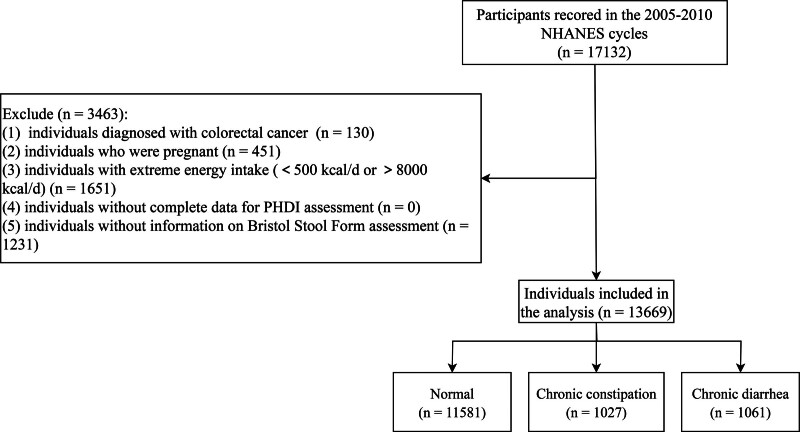
The screening flowchart of the study populations.

The 2005 to 2010 NHANES cycles included 17,132 participants aged ≥20 years. Following screening, 3463 participants were excluded, with 13,669 eligible individuals retained for analysis (Fig. 1). Participant characteristics are summarized in Table 1. The mean age of the participants was 46.66 (±0.35) years and 6725 (50.62%) participants were female. Participants showed a mean PHDI score of 65.86 (±0.07). Among participants, 1027 reported chronic constipation, 1061 had chronic diarrhea, and 11,581 maintained normal bowels. Significant differences emerged in age, gender, race, education, PIR, current drinking, physical activity, hypertension, diabetes, dyslipidemia, CVD, CKD, depression, WHR, and total energy when comparing normal bowels with chronic constipation and diarrhea groups (*P* < .05).

**Table 1:** Characteristics of participants.

Variables	Total (N = 13669)	Normal (N = 11581)	Chronic constipation (N = 1027)	Chronic diarrhea (N = 1061)	*P*
PHDI, score, Mean (±SE)	65.86 (±0.07)	65.92 (±0.07)	65.01 (±0.17)	65.90 (±0.19)	<.001
PHDI, n (%)					<.001
<63.69	4556 (31.09)	3755 (30.09)	419 (41.53)	382 (32.88)	
63.69–66.67	4557 (33.57)	3898 (34.14)	318 (30.16)	341 (29.78)	
>66.67	4556 (35.35)	3928 (35.77)	290 (28.31)	338 (37.34)	
Age, years, Mean (±S.E)	46.66 (±0.35)	46.50 (±0.37)	45.55 (±0.73)	49.83 (±0.70)	<.001
Gender, n (%)					<.001
Male	6944 (49.38)	6148 (51.58)	339 (29.59)	457 (41.94)	
Female	6725 (50.62)	5433 (48.42)	688 (70.41)	604 (58.06)	
Race, n (%)					<.001
Non-Hispanic White	6805 (71.02)	5900 (72.02)	447 (63.11)	458 (66.58)	
Non-Hispanic Black	2712 (11.22)	2247 (10.66)	241 (15.88)	224 (13.53)	
Mexican American	2459 (8.25)	2041 (7.98)	189 (10.14)	229 (9.66)	
Other race	1693 (9.51)	1393 (9.34)	150 (10.87)	150 (10.22)	
Education, n (%)					<.001
High school and below	7067 (42.33)	5781 (40.83)	622 (53.44)	664 (49.85)	
Above high school	6602 (57.67)	5800 (59.17)	405 (46.56)	397 (50.15)	
PIR, n (%)					.003
<1	2435 (12.40)	1970 (11.85)	216 (15.51)	249 (16.08)	
≥1	10,255 (82.01)	8803 (82.76)	734 (77.90)	718 (76.70)	
Unknown	979 (5.60)	808 (5.39)	77 (6.59)	94 (7.22)	
Marriage, n (%)					.088
Married	8346 (63.25)	7134 (63.64)	572 (58.53)	640 (63.21)	
No married	5323 (36.75)	4447 (36.36)	455 (41.47)	421 (36.79)	
Current smoking, n (%)					.067
No	10,603 (76.96)	8983 (77.14)	823 (78.79)	797 (72.69)	
Yes	3066 (23.04)	2598 (22.86)	204 (21.21)	264 (27.31)	
Current drinking, n (%)					<.001
No	3782 (23.20)	3046 (22.01)	392 (33.64)	344 (27.46)	
Yes	9887 (76.80)	8535 (77.99)	635 (66.36)	717 (72.54)	
Physical activity, MET·min/wk, n (%)					<.001
<600	2192 (16.95)	1853 (16.81)	171 (18.05)	168 (17.62)	
≥600	7289 (57.89)	6321 (59.04)	483 (51.76)	485 (49.72)	
Unknown	4188 (25.16)	3407 (24.16)	373 (30.18)	408 (32.66)	
Hypertension, n (%)					.011
No	6087 (49.23)	5212 (49.39)	506 (52.63)	369 (43.71)	
Yes	7582 (50.77)	6369 (50.61)	521 (47.37)	692 (56.29)	
Diabetes, n (%)					<.001
No	11,254 (87.63)	9607 (88.08)	856 (88.91)	791 (80.57)	
Yes	2415 (12.37)	1974 (11.92)	171 (11.09)	270 (19.43)	
Dyslipidemia, n (%)					.024
No	3919 (29.47)	3359 (29.72)	324 (31.27)	236 (24.44)	
Yes	9750 (70.53)	8222 (70.28)	703 (68.73)	825 (75.56)	
CVD, n (%)					.002
No	11,354 (87.01)	9691 (87.41)	837 (86.73)	826 (82.21)	
Yes	2315 (12.99)	1890 (12.59)	190 (13.27)	235 (17.79)	
CKD, n (%)					<.001
No	11,345 (87.59)	9704 (88.17)	840 (85.64)	801 (82.35)	
Yes	2324 (12.41)	1877 (11.83)	187 (14.36)	260 (17.65)	
Depression, n (%)					<.001
No	12,493 (92.46)	10,722 (93.58)	890 (86.10)	881 (84.85)	
Yes	1176 (7.54)	859 (6.42)	137 (13.90)	180 (15.15)	
WHR, ratio, Mean (±SE)	0.58 (±0.00)	0.58 (±0.00)	0.57 (±0.00)	0.61 (±0.00)	<.001
Total energy, kcal, Mean (±SE)	2033.07 (±14.80)	2055.40 (±15.68)	1816.09 (±29.54)	1975.11 (±41.05)	<.001

CKD = chronic kidney disease, CVD = cardiovascular disease, PHDI = the Planetary Health Diet Index, PIR = poverty-to-income ratio, SE = standard errors, WHR = waist-to-height ratio.

### 3.2. Relationship between PHDI and chronic constipation/diarrhea

The correlation between PHDI and chronic constipation/diarrhea is listed in Table 2. Higher PHDI scores had a connection with lower odds of chronic constipation (OR [95% CI] = 0.93 [0.90–0.96], *P* < .001), though no significant correlation emerged with chronic diarrhea (OR = 1.00 [0.97–1.03], *P* = .894). After confounder adjustment, higher PHDI scores continued to correlate with lower odds of chronic constipation (OR = 0.95 [0.92–0.98], *P* = .001). When PHDI was categorized by tertiles, the second (63.69–66.67) and third (>66.67) tertiles of PHDI were linked to lower odds of chronic constipation versus the first tertile (<63.69) in both unadjusted (63.69–66.67: OR = 0.64 [0.51–0.80], *P* < .001; >66.67: OR = 0.57 [0.46–0.71], *P* < .001) and adjusted (63.69–66.67: OR = 0.73 [0.57–0.92], *P* = .009; >66.67: 0.68 [0.54–0.85], *P* = .001) analyses.

**Table 2:** The correlation between PHDI and chronic constipation/diarrhea in the general population.

Outcome	Variables	Univariable analysis		Multivariable analysis	
		OR (95% CI)	*P*	OR (95% CI)	*P*
Chronic constipation	PHDI	0.93 (0.90–0.96)	<.001	0.95 (0.92–0.98)	.001
	PHDI				
	<63.69	Ref		Ref	
	63.69–66.67	0.64 (0.51–0.80)	<.001	0.73 (0.57–0.92)	.009
	>66.67	0.57 (0.46–0.71)	<.001	0.68 (0.54–0.85)	.001
Chronic diarrhea	PHDI	1.00 (0.97–1.03)	.894	1.02 (0.99–1.05)	.268
	PHDI				
	<63.69	Ref		Ref	
	63.69–66.67	0.80 (0.64–0.99)	.045	0.88 (0.70–1.10)	.243
	>66.67	0.96 (0.78–1.18)	.661	1.09 (0.87–1.37)	.446

Multivariable logistic regression analyses adjusted for chronic constipation: gender, race, education, PIR, marriage, current drinking, depression, and total energy; and chronic diarrhea: age, gender, race, education, PIR, current smoking, current drinking, physical activity, hypertension, diabetes, dyslipidemia, CVD, CKD, depression, and WHR.

CI = confidence interval, CKD = chronic kidney disease, CVD = cardiovascular disease, OR = odds ratio, PHDI = the Planetary Health Diet Index, PIR = poverty-to-income ratio, Ref = reference, WHR = waist-to-height ratio.

**Figure 2.**
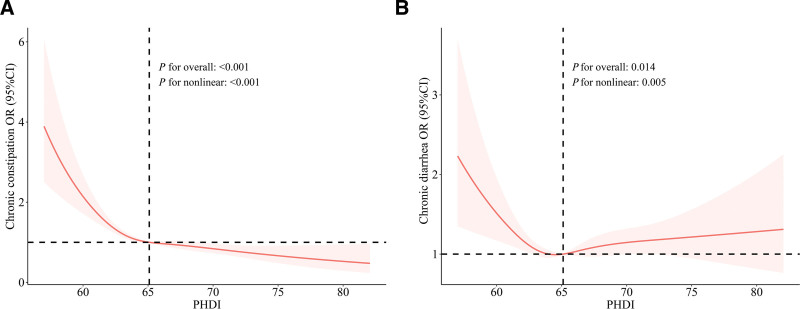
The nonlinear correlation of PHDI score with chronic constipation/diarrhea risk. (A) The correlation between PHDI and chronic constipation. (B) The correlation between PHDI and chronic diarrhea. Risk of chronic constipation or diarrhea varies with PHDI values. PHDI = the Planetary Health Diet Index.

Figure 2 presents the nonlinear correlation of PHDI with chronic constipation/diarrhea. There was a nonlinear link of PHDI with chronic constipation (*P*_overall_ < .001, *P*_nonlinear_ < .001) and chronic diarrhea (*P*_overall_ = .014, *P*_nonlinear_ = .005). The risk of chronic constipation in individuals decreased with the increase in PHDI (Fig. 2A), and the relationship between PHDI and chronic diarrhea was “L” shaped (Fig. 2B).

**Figure 3.**
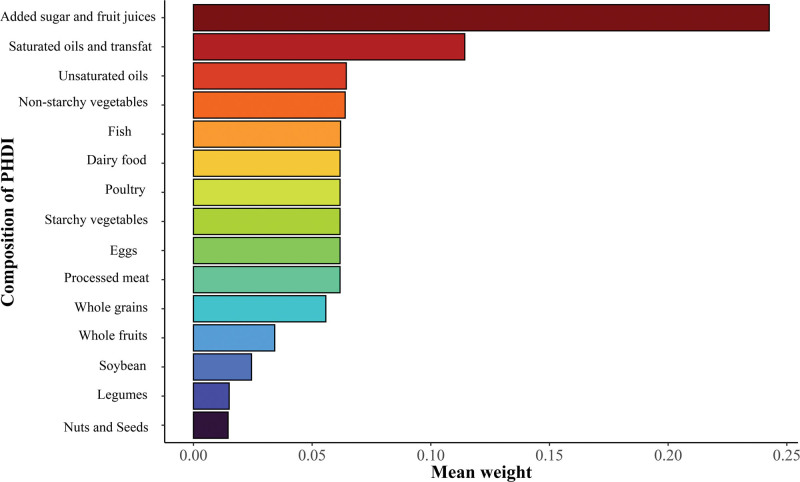
The importance of each component in PHDI score. The larger the mean weight value, the greater the contribution to PHDI. PHDI = the Planetary Health Diet Index.

The WQS analysis indicated that the overall mixed exposure effect of PHDI on the risk of chronic constipation was 0.96 (95% CI: 0.94–0.97, *P* < .001), demonstrating that the overall mixed exposure effect of PHDI was linked to a reduced risk of chronic constipation. However, no correlation was found between the overall mixed exposure effect of PHDI and chronic diarrhea risk (OR = 1.00 [0.98–1.02], *P* = .871). Among the contributions of each component to PHDI, “added sugar and fruit juices” contributed the most to the effect of PHDI, followed by “saturated oils and transfat,” while “legumes” and “nuts and seeds” contributed the least (Fig. 3).

### 3.3. Subgroup analyses of the correlation between PHDI and chronic constipation

**Figure 4.**
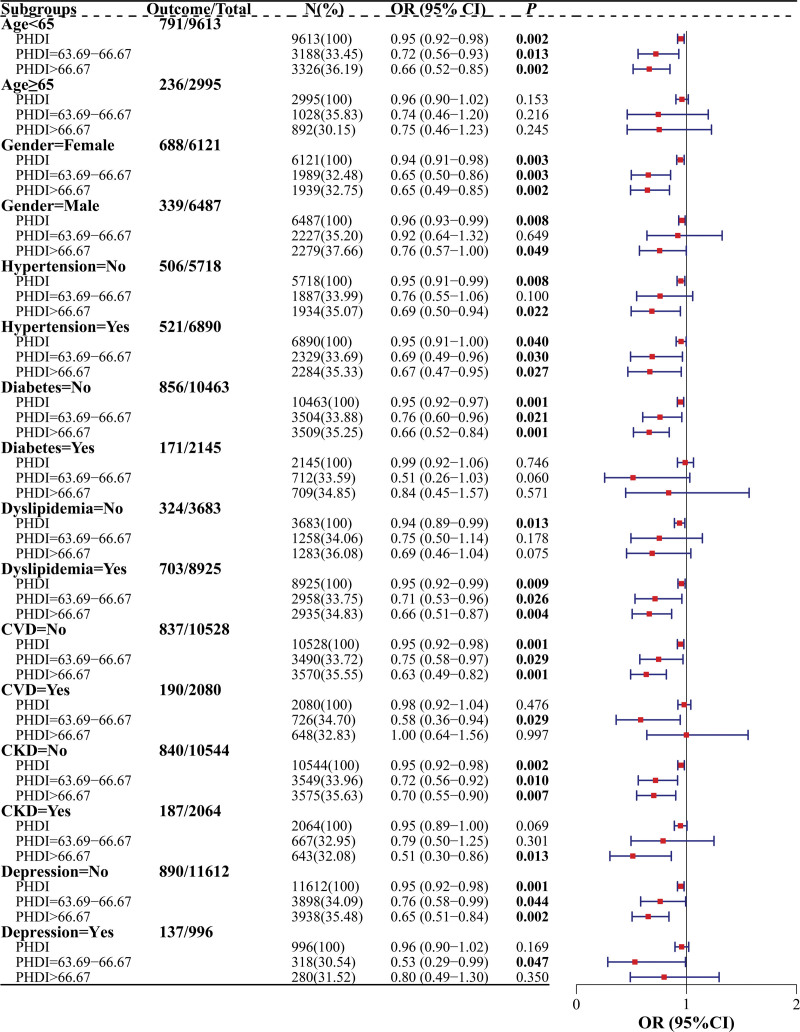
The correlation between PHDI and chronic constipation of different characteristics subgroups. CKD = chronic kidney disease, CVD = cardiovascular disease, PHDI = the Planetary Health Diet Index.

The correlation between PHDI and chronic constipation of age, gender, hypertension, diabetes, dyslipidemia, CVD, CKD, and depression subgroups is listed in Figure 4. PHDI values of 63.69 to 66.67 (OR = 0.72 [0.56–0.93], *P* = .013) and >66.67 (OR = 0.66 [0.52–0.85], *P* = .002) were connected with a lower risk of chronic constipation in individuals aged <65 years, but not in individuals aged ≥65years (*P* > .05). For gender subgroups, PHDI values of 63.69 to 66.67 (OR = 0.65 [0.50–0.86], *P* = .003) and >66.67 (OR = 0.65 [0.49–0.85], *P* = .002) were linked to a lower risk of chronic constipation in females, and PHDI values of >66.67 (OR = 0.76 [0.57–1.00], *P* = .049) was also related to a lower risk of chronic constipation in males. Among hypertension subgroups, PHDI values of 63.69 to 66.67 (OR = 0.69 [0.49–0.96], *P* = .030) and >66.67 (OR = 0.67 [0.47–0.95], *P* = .027) were associated with a lower risk of chronic constipation in individuals with hypertension, and PHDI values of >66.67 (OR = 0.69 [0.50–0.94], *P* = .022) was linked to a lower risk of chronic constipation in individuals without hypertension. In diabetes subgroups, PHDI values of 63.69 to 66.67 (OR = 0.76 [0.60–0.96], *P* = .021) and >66.67 (OR = 0.66 [0.52–0.84], *P* = .001) were related to a lower risk of chronic constipation in individuals without diabetes, but not in individuals with diabetes (*P* > .05). For dyslipidemia subgroups, PHDI values of 63.69 to 66.67 (OR = 0.71 [0.53–0.96], *P* = .026) and >66.67 (OR = 0.66 [0.51–0.87], *P* = .004) were connected to a lower risk of chronic constipation in individuals with dyslipidemia, but not in individuals without dyslipidemia (*P* > .05). Among CVD subgroup, PHDI values of 63.69 to 66.67 (OR = 0.75 [0.58–0.97], *P* = .029) and >66.67 (OR = 0.63 [0.49–0.82], *P* = .001) were linked to a lower risk of chronic constipation in individuals without CVD, and PHDI values of 63.69 to 66.67 (OR = 0.58 [0.36–0.94], *P* = .029) were also associated with a lower risk of chronic constipation in individuals with CVD. For CKD subgroup, PHDI values of 63.69 to 66.67 (OR = 0.72 [0.56–0.92], *P* = .010) and >66.67 (OR = 0.70 [0.55–0.90], *P* = .007) were related to a lower risk of chronic constipation in individuals without CKD, and PHDI values of >66.67 (OR = 0.51 [0.30–0.86], *P* = .013) were linked to a lower risk of chronic constipation in individuals with CKD. Among depression subgroups, PHDI values of 63.69 to 66.67 (OR = 0.76 [0.58–0.99], *P* = .044) and >66.67 (OR = 0.65 [0.51–0.84], *P* = .002) were connected to a lower risk of chronic constipation in individuals without depression, and PHDI values of 63.69 to 66.67 (OR = 0.53 [0.29–0.99], *P* = .047) were also connected to a lower risk of chronic constipation in individuals with depression.

## 4. Discussion

This study examined the connection between PHDI, which represents a healthy diet, and the risk of chronic constipation and diarrhea in the general population. The analysis revealed that high PHDI scores had a connection with a reduced risk of chronic constipation, but no link between PHDI and chronic diarrhea was observed. PHDI demonstrated a nonlinear correlation with chronic constipation risk. Further analysis indicated that the connection between PHDI and chronic constipation risk was more obvious in populations aged <65 years, those without diabetes, and those with dyslipidemia.

The pathogenesis of constipation is multifactorial, and the main factors in the development of chronic constipation include diet, lifestyle factors, and primary disturbances in bowel function due to impaired colonic advancement or rectal emptying.^[4]^ Diet is a common and modifiable factor influencing the occurrence of chronic constipation. Studies on the impact of diet on constipation have focused on the role of dietary fiber,^[18,29]^ but the combined effects of complex other components in the diet have been less reported. The PHDI systematically assesses daily diets based on 15 food groups, with higher PHDI scores representing healthier diets. The current study examined the link of PHDI with chronic constipation and diarrhea risk in the general population. In our findings, high PHDI scores were connected to a lower risk of chronic constipation, but no connection between PHDI and chronic diarrhea was identified. Previous studies have found that high PHDI scores were connected to a reduced risk of CVD, metabolic abnormalities, and fatty liver,^[12,13,15]^ but no studies have explored the link of PHDI scores with chronic constipation and diarrhea. The correlation between a high PHDI score and a reduced risk of chronic constipation may be linked to the following reasons. High-fiber diets increase fecal weight, resulting in shorter colonic transit time, which reduces the risk of constipation,^[30]^ and this ameliorative effect may be caused primarily by soluble fiber.^[31]^ High PHDI scores improved gut microbiota diversity as well as reduced gut microbiota-related inflammation.^[16,17]^ A high PHDI was connected to a reduced risk of obesity,^[32,33]^ and obesity was in turn correlated with an increased risk of chronic constipation.^[24,34]^ PHDI may improve constipation by reducing the risk of obesity. Moreover, there is a connection between dietary patterns, gut microbiota, and metabolic diseases. Dietary patterns influence changes in the gut microbiota, while gut microbiota and their metabolites contribute to metabolic disorders through alterations in intestinal permeability, immune responses, inflammatory responses, and insulin resistance.^[35,36]^

Our analysis also found that mixed exposure to the 15 components of PHDI was also correlated with a reduced risk of chronic constipation. In the analysis of the contributions of these 15 components to PHDI, “added sugar and fruit juices” contributed the most to PHDI, followed by “saturated oils and transfat,” “unsaturated oils,” and “non-starchy vegetables,” while “legumes” and “nuts and seeds” contributed the least. “Added sugar and fruit juices” represents sugary drinks, which are usually high in sugar and calories. Increased consumption of sugary drinks has been found to be connected to an increased risk of constipation.^[37]^ Lower intakes of saturated oils and transfat corresponded to higher scores of PHDI fractions, whereas higher intakes of unsaturated oils corresponded to higher scores of PHDI fractions. Saturated fatty acids have pro-inflammatory effects,^[38]^ and trans fatty acids exacerbate the pathological inflammation of the intestinal epithelium in individuals with inflammatory bowel disease.^[39]^ Higher intakes of saturated fatty acids and trans fatty acids are correlated with an increased risk of ulcerative colitis.^[40]^ For unsaturated oils, a randomized controlled trial demonstrated that intake of flaxseed flour (rich in various unsaturated fatty oils) significantly improved bowel movement frequency and severity of abdominal pain in patients with constipation versus those taking lactulose.^[41]^ For vegetables, non-starchy vegetables (especially tomatoes) were connected to a reduced risk of constipation, and the risk of constipation was not affected by the intake of starchy vegetables (e.g., potatoes).^[42]^ These findings may provide recommendations for daily diets, specifically that the intake of added sugar beverages and saturated oils should be minimized and the intake of unsaturated oils and non-starchy vegetables should be increased. Additionally, the global burden of irritable bowel syndrome may be increasing due to the spread of Westernized diets high in sugar and fat and changes in lifestyle worldwide.^[43]^ Against this global backdrop, individuals stand to gain potential benefits from dietary changes based on a high PHDI diet.

This investigation is the first to examine the correlation between PHDI and chronic constipation and diarrhea. Through various analytical methods, we ensured the reliability of the findings. Moreover, the data for the analysis were obtained from the nationally representative NHANES database, which demonstrates the generalizability of the results. However, several limitations should be noted. First, bowel status in the NHANES survey was collected by questionnaire, which may be affected by recall bias. Second, the dietary data in NHANES were obtained through 24-hour recall interviews, which are inevitably subject to recall bias. Whether these dietary data can represent the long-term dietary patterns of the participants also needs to be verified. Third, the data analyzed in this study were cross-sectional and could not be assessed for a causal relationship between PHDI and chronic constipation.

## 5. Conclusions

High PHDI scores were correlated with a reduced risk of chronic constipation, but not correlated with chronic diarrhea. PHDI presented a nonlinear relationship with chronic constipation risk. These findings suggest that dietary patterns may influence metabolic health. Individuals may benefit from a diet that reduces the intake of added sugar drinks and saturated oils as well as increases the intake of unsaturated oils and non-starchy vegetables.

## Author contributions

**Conceptualization:** Renwei Qi, Sinan Xu.

**Data curation:** Renwei Qi, Haoliang Zhai, Dongjia Xu, Yu Wen, Sinan Xu.

**Methodology:** Haoliang Zhai, Dongjia Xu, Yu Wen.

**Supervision:** Sinan Xu.

**Validation:** Haoliang Zhai, Dongjia Xu, Yu Wen.

**Writing – original draft:** Renwei Qi.

**Writing – review & editing:** Renwei Qi, Sinan Xu.

## Supplementary Material


