# Supplementary material for: Association between the Planetary Health Diet Index and chronic constipation and diarrhea risk in general population: A cross-sectional analysis of NHANES
Source: Medicine (Baltimore). 2025 Nov 7;104(45):e45468. doi: 10.1097/MD.0000000000045468 (PMC12599696; doi:10.1097/MD.0000000000045468)
Supplement: Supplementary file 1 [file medi-104-e45468-s001.docx]

**Supplement Figure 1**. Premise assumptions testing of the logistic regression model.


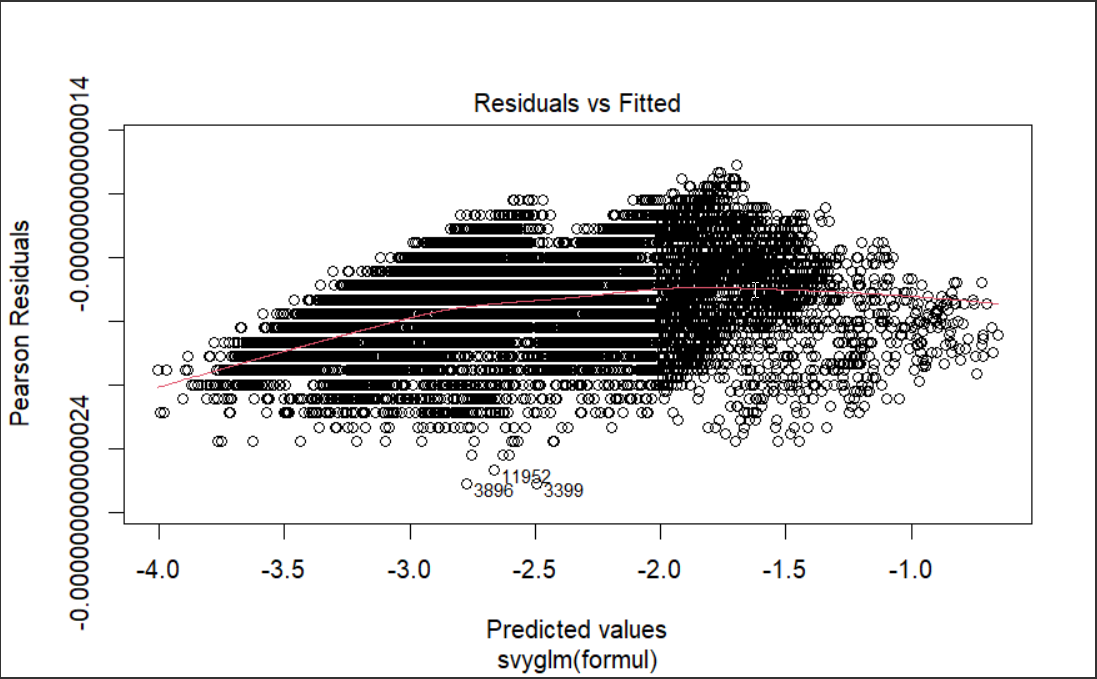

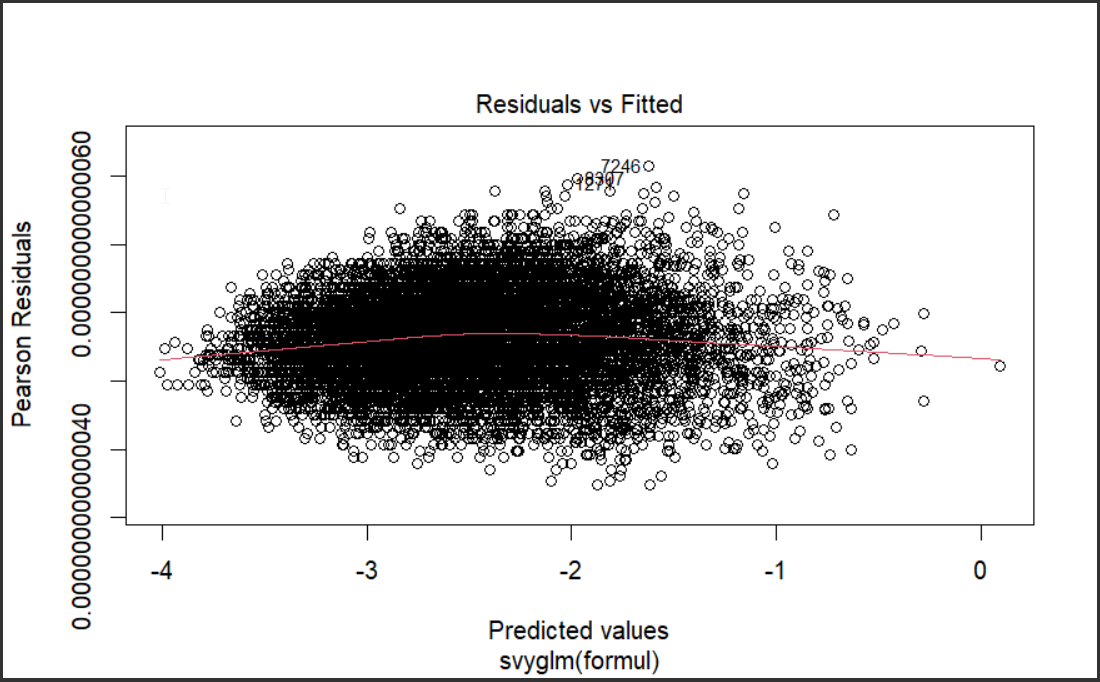


(chronic constipation) (chronic diarrhea)

**(A)** Linearity. The residual values exhibit a relatively balanced distribution compared to the fitted values, with the fitted line appearing relatively stable. No significant fluctuations in residual values were observed as predicted values changed. Therefore, the model can be considered linear.

| Outcomes | Variables | VIF | Df | VIF^(1/(2*Df)) |
| --- | --- | --- | --- | --- |
| Chronic constipation | Gender | 1.768 | 1 | 1.329 |
|  | Race | 1.652 | 3 | 1.087 |
|  | Education | 1.289 | 1 | 1.135 |
|  | PIR | 2.422 | 2 | 1.248 |
|  | Marriage | 1.558 | 1 | 1.248 |
|  | Drinking | 1.831 | 1 | 1.353 |
|  | Depression | 1.893 | 1 | 1.376 |
|  | Total energy | 1.324 | 1 | 1.15 |
|  |  |  |  |  |
| Chronic diarrhea | Age | 3.943 | 1 | 1.986 |
|  | Gender | 2.433 | 1 | 1.56 |
|  | Race | 7.403 | 3 | 1.396 |
|  | Education | 2.545 | 1 | 1.595 |
|  | PIR | 4.451 | 2 | 1.452 |
|  | Smoking | 2.624 | 1 | 1.62 |
|  | Drinking | 4.567 | 1 | 2.137 |
|  | Physical activity | 3.437 | 2 | 1.362 |
|  | Hypertension | 4.719 | 1 | 2.172 |
|  | Diabetes | 4.25 | 1 | 2.062 |
|  | Dyslipidemia | 3.555 | 1 | 1.885 |
|  | CVD | 3.34 | 1 | 1.827 |
|  | CKD | 3.718 | 1 | 1.928 |
|  | Depression | 2.799 | 1 | 1.673 |
|  | WHtR | 3.757 | 1 | 1.938 |

Note: VIF, variance inflation factor.

**(B)** Independence among independent variables. The VIF values for all variables are below 3, indicating no multicollinearity exists among the variables in the model.

| Models | X-squared | P |
| --- | --- | --- |
| Chronic constipation | 550.41 | 0.0519 |
| Chronic diarrhea | 6.3339 | 0.09645 |

**(C)** The Hosmer-Lemeshow test for the model goodness-of-fit. A P > 0.05 indicates that the model passes the test.

**Supplement Table 1**. The intake criterion for the 15 food groups in the PHDI.

| Component | Min score (0) | Max score (10) | Weight |
| --- | --- | --- | --- |
| Whole grains (grams/day) |  |  |  |
| Women | 0 | ≥75 | 1 |
| Men | 0 | ≥90 | 1 |
| Starchy vegetables (grams/day) | ≥200 | ≤50 | 1 |
| Non-starchy vegetables (grams/day) | 0 | ≥300 | 1 |
| Whole fruits (grams/day) | 0 | ≥200 | 1 |
| Dairy food (grams/day) | ≥1000 | ≤250 | 1 |
| Red/processed meat (grams/day) | ≥100 | ≤14 | 1 |
| Poultry (grams/day) | ≥100 | ≤29 | 1 |
| Eggs (grams/day) | ≥120 | ≤13 | 1 |
| Fish (grams/day) | 0 | ≥28 | 1 |
| Nuts and seeds (grams/day) | 0 | ≥50 | 1 |
| Legumes (grams/day) | 0 | ≥100 | 0.5 |
| Soybean (grams/day) | 0 | ≥50 | 0.5 |
| Saturated oils and transfat (% of total energy intake) | ≥10% | 0 | 1 |
| Unsaturated oils (% of total energy intake) | ≤3.5% | ≥21% | 1 |
| Added sugar and fruit juices (% of total energy intake) | ≥25% | ≤5% | 1 |

Note: PHDI, the Planetary Health Diet Index.

**Supplement Table 2**. Differences between pre- and post-imputation for missing variables.

| Variables | Pre-imputation | Post-imputation | Statistics | *P* |
| --- | --- | --- | --- | --- |
| Depression, Mean (±S.E) | 3.00 (±0.07) | 3.00 (±0.07) | t = -0.991 | 0.327 |
| Current smoking, n (%) |  |  | χ² = 2.135 | 0.151 |
| No | 10600 (76.95) | 10603 (76.96) |  |  |
| Yes | 3066 (23.05) | 3066 (23.04) |  |  |
| Marriage, n (%) |  |  | χ² = 0.132 | 0.718 |
| Married | 8341 (63.25) | 8346 (63.25) |  |  |
| No married | 5321 (36.75) | 5323 (36.75) |  |  |
| Education, n (%) |  |  | χ² = 0.073 | 0.788 |
| High School and below | 7063 (42.33) | 7067 (42.33) |  |  |
| Above high school | 6596 (57.67) | 6602 (57.67) |  |  |
| Current drinking, n (%) |  |  | χ² = 0.000 | 0.995 |
| No | 3778 (23.20) | 3782 (23.20) |  |  |
| Yes | 9880 (76.80) | 9887 (76.80) |  |  |
| Height, Mean (±S.E) | 169.10 (±0.13) | 169.09 (±0.13) | t = -1.548 | 0.129 |
| Waist circumference, Mean (±S.E) | 98.09 (±0.34) | 98.10 (±0.34) | t = 1.280 | 0.207 |

**Supplement Table 3**. Confounding factors related to chronic constipation/diarrhea through weighted univariable logistic regression.

| Variables | Chronic constipation | | Chronic diarrhea | |
| --- | --- | --- | --- | --- |
|  | OR (95% CI) | *P* | OR (95% CI) | *P* |
| Age | 1.00 (0.99-1.00) | 0.206 | 1.01 (1.01-1.02) | <0.001 |
| Gender |  |  |  |  |
| Male | Ref |  | Ref |  |
| Female | 2.53 (2.04-3.15) | <0.001 | 1.47 (1.24-1.76) | <0.001 |
| Race |  |  |  |  |
| Non-Hispanic White | Ref |  | Ref |  |
| Non-Hispanic Black | 1.70 (1.34-2.16) | <0.001 | 1.37 (1.12-1.69) | 0.003 |
| Mexican American | 1.45 (1.07-1.96) | 0.016 | 1.31 (0.96-1.78) | 0.083 |
| Other Race | 1.33 (0.94-1.87) | 0.100 | 1.18 (0.85-1.65) | 0.312 |
| Education |  |  |  |  |
| High School and below | Ref |  | Ref |  |
| Above high school | 0.60 (0.51-0.71) | <0.001 | 0.69 (0.58-0.84) | <0.001 |
| PIR |  |  |  |  |
| <1 | Ref |  | Ref |  |
| ≥1 | 0.72 (0.59-0.88) | 0.002 | 0.68 (0.53-0.88) | 0.004 |
| Unknown | 0.93 (0.61-1.44) | 0.751 | 0.99 (0.63-1.55) | 0.956 |
| Marriage |  |  |  |  |
| Married | Ref |  | Ref |  |
| No married | 1.24 (1.02-1.51) | 0.033 | 1.02 (0.84-1.24) | 0.851 |
| Current smoking |  |  |  |  |
| No | Ref |  | Ref |  |
| Yes | 0.91 (0.74-1.12) | 0.367 | 1.27 (1.01-1.60) | 0.045 |
| Current drinking |  |  |  |  |
| No | Ref |  | Ref |  |
| Yes | 0.56 (0.45-0.68) | <0.001 | 0.75 (0.61-0.91) | 0.004 |
| Physical activity |  |  |  |  |
| <600 | Ref |  | Ref |  |
| ≥600 | 0.82 (0.62-1.07) | 0.135 | 0.80 (0.65-0.99) | 0.040 |
| Unknown | 1.16 (0.89-1.53) | 0.269 | 1.29 (0.98-1.71) | 0.074 |
| Hypertension |  |  |  |  |
| No | Ref |  | Ref |  |
| Yes | 0.88 (0.73-1.05) | 0.158 | 1.26 (1.06-1.49) | 0.009 |
| Diabetes |  |  |  |  |
| No | Ref |  | Ref |  |
| Yes | 0.92 (0.73-1.16) | 0.485 | 1.78 (1.46-2.18) | <0.001 |
| Dyslipidemia |  |  |  |  |
| No | Ref |  | Ref |  |
| Yes | 0.93 (0.79-1.09) | 0.369 | 1.31 (1.07-1.60) | 0.011 |
| CVD |  |  |  |  |
| No | Ref |  | Ref |  |
| Yes | 1.06 (0.86-1.31) | 0.564 | 1.50 (1.18-1.91) | 0.001 |
| CKD |  |  |  |  |
| No | Ref |  | Ref |  |
| Yes | 1.25 (0.98-1.60) | 0.074 | 1.60 (1.30-1.96) | <0.001 |
| Depression |  |  |  |  |
| No | Ref |  | Ref |  |
| Yes | 2.35 (1.71-3.25) | <0.001 | 2.60 (1.90-3.56) | <0.001 |
| WHR | 0.40 (0.13-1.25) | 0.113 | 31.44 (12.28-80.49) | <0.001 |
| Total energy | 1.00 (1.00-1.00) | <0.001 | 1.00 (1.00-1.00) | 0.056 |

Note: OR, odds ratio; CI, confidence interval; Ref, reference; PIR, poverty-to-income ratio; CVD, cardiovascular disease; CKD, chronic kidney disease; WHR, waist-to-height ratio.
